# Do matrix metalloproteinase and cathepsin K inhibitors work synergistically to reduce dentin erosion?

**DOI:** 10.1590/1678-7757-2022-0449

**Published:** 2023-05-08

**Authors:** Xiujiao LIN, Xinwen TONG, Hui YANG, Yiying CHEN, Hao YU

**Affiliations:** 1 Fujian Medical University School and Hospital of Stomatology Fujian Key Laboratory of Oral Diseases & Fujian Provincial Engineering Research Fuzhou China Fujian Medical University, School and Hospital of Stomatology, Fujian Key Laboratory of Oral Diseases & Fujian Provincial Engineering Research Center of Oral Biomaterial & Stomatological Key Laboratory of Fujian College and University, Fuzhou, China.; 2 Fujian Medical University Department of Prosthodontics & Research Center of Dental Esthetics and Biomechanics Fuzhou China Fujian Medical University, Department of Prosthodontics & Research Center of Dental Esthetics and Biomechanics, Fuzhou, China.; 3 Tohoku University Graduate School of Dentistry Liaison Center for Innovative Dentistry Sendai Japan Tohoku University, Graduate School of Dentistry, Liaison Center for Innovative Dentistry, Sendai, Japan.; 4 Nagasaki University Graduate School of Biomedical Sciences Department of Applied Prosthodontics Nagasaki Japan Nagasaki University, Graduate School of Biomedical Sciences, Department of Applied Prosthodontics, Nagasaki, Japan.

**Keywords:** Dentin erosion, Matrix metalloproteinase, Cathepsin K, Inhibitors

## Abstract

**Objectives:**

To evaluate the effects of matrix metalloproteinase (MMP) and cathepsin K (catK) inhibitors on resistance to dentin erosion.

**Methodology:**

A total of 96 dentin specimens (3×3×2 mm) were prepared and randomly assigned into four groups (n=24): deionized water (DW); 1 µM odanacatib (ODN, catK inhibitor); 1 mM 1,10-phenanthroline (PHEN, MMP inhibitor); and 1 µM odanacatib + 1 mM 1,10-phenanthroline (COM). Each group was further divided into two subgroups for the application of treatment solutions before (PRE) and after erosive challenges (POST). All specimens were subjected to four daily erosive challenges for 5 d. For each erosive challenge, the specimens in subgroup PRE were immersed in the respective solutions before cola drinks, while the specimens in subgroup POST were immersed in the respective solutions after cola drinks (the immersion duration was 5 min in both cases). All specimens were stored in artificial saliva at 37°C between erosive challenges. The erosive dentin loss (EDL) was measured by profilometry. The residual demineralized organic matrix (DOM) of specimens was removed using type VII collagenase and evaluated by profilometry. Both the EDL and thickness of the residual DOM were statistically analyzed by two-way analysis of variance (ANOVA) and Bonferroni’s test (α=0.05). The surface topography and transverse sections of the specimens were observed using SEM. MMPs and catK were immunolabeled in the eroded dentin and in situ zymography was performed to evaluate the enzyme activity.

**Results:**

Significantly lower EDL was found in the groups ODN, PHEN, and COM than in the control group (all p<0.05), while no significant difference in EDL was found among the groups ODN, PHEN, and COM (all p>0.05). The application sequence showed no significant effect on the EDL of the tested groups (p=0.310). A significantly thicker DOM was observed in the group ODN than in the control group regardless of the application sequence (both p<0.05). The treatment with ODN, PHEN, and COM inhibited the gelatinolytic activity by approximately 46.32%, 58.6%, and 74.56%, respectively.

**Conclusions:**

The inhibition of endogenous dentinal MMPs and catK increases the acid resistance of human dentin but without an apparent synergistic effect. The inhibition of MMPs and catK is equally effective either before or after the acid challenge.

## Introduction

Tooth erosion occurs by the dissolution of dental apatite when exposed to acids, without bacterial involvement,^1^ with a global prevalence in adolescence estimated at 30%.^2^ For tooth erosion in the initial stage, the effects of acids are limited to the enamel.^3^ Exposure to acids results in the dissolution of the inorganic dental hard tissue,^3^ which progressively leads to extensive loss of substance.^3^ When enamel loss occurs, the effects of acids further extend into the dentin, and dentin erosion can occur.^3,4^ During the progression of dentin erosion, minerals are released from the peritubular and intertubular dentin.^5^ Thereafter, a layer of a fully demineralized organic matrix (DOM) is formed in the superficial dentin, followed by a partially demineralized zone and by the intact inner dentin.^4,5^ DOM is regarded as a diffusion barrier against erosive acids and active ingredients released by demineralization, as well as a form of resistance to abrasive forces.^6,7^ Furthermore, maintaining DOM is important for dentin remineralization, especially in the presence of calcium and phosphate together with fluoride.^8^ However, DOM can be degraded by collagenases (e.g., matrix metalloproteinases (MMPs) and cysteine cathepsins (CCs)), leading to increased mineral loss in demineralized dentin and further accelerating the progression of dentin erosion.^8-10^ In previous studies, the application of both MMP^5,11^ and CC inhibitors^5^ significantly reduced erosive dentin loss (EDL) by approximately 21–42%.

MMPs are mostly secreted as latent proenzymes (stated as proMMPs), in which the functional activity of the catalytic domain is inhibited by the prodomain.^12^ When the prodomain is removed by other proteases, such as CCs, proMMPs can be activated.^12^ Christensen and Shastri^13^ (2015) reported that proMMP-9 was activated because the prodomain was cleaved by cathepsin K (catK) *in vitro*. Moreover, using odanacatib (a catK inhibitor) significantly reduced the release of cross-linked carboxyterminal telopeptide of type I collagen (ICTP, the specific products of type I collagen degraded by MMPs).^14^ On the other hand, Li, et al.^15^ (2004) found that MMP-1 cleaved type I collagen released 3/4 and 1/4 fragments, which allowed further degradation by CCs (e.g., catK, catB, and catL). Therefore, MMPs and CCs may synergistically contribute to the progression of dentin erosion.^5,13,15^ In our previous study, 1,10-phenanthroline (an MMP inhibitor), E-64 (a CC inhibitor), and a combination of the two inhibitors were applied before erosion, aiming to reduce EDL.^5^ In that study, EDL was significantly reduced and a thicker DOM was preserved compared to that of the control group.^5^ However, no significant differences were found either in EDL or DOM between individual and combined applications.^5^ This phenomenon may be related to the application sequence of MMP and catK inhibitors. Importantly, MMPs become functional after the acidic pH is neutralized by the saliva buffer, while CCs exhibit optimal functional activity at a slightly acidic pH (e.g., catK exhibits optimal activity at pH=5.5) and may be irreversibly inactivated at a neutral pH.^11^ Moreover, a recent study showed that chlorhexidine performed better than sodium fluoride in controlling dentin erosion and, interestingly, chlorhexidine was more effective in reducing EDL when applied after erosion than when applied before erosion. Applying chlorhexidine to patients at risk of dentin erosion (e.g., in the event of gingival recession) before an acid invasion can be considered a pre-erosion application, whereas applying chlorhexidine to patients suffering from dentin erosion can be considered a post-erosion application.^16^ It was also reported that sodium fluoride could inhibit MMPs and that chlorhexidine could inhibit MMPs as well as CCs.^17,18^ Accordingly, a synergistic effect of MMP and CC inhibitors applied after erosion may exist. Furthermore, erosive attacks would result in a hollowing and funneling of the dentinal tubule.^19^ Compared to prior treatment with an inhibitor, prior treatment with acid may increase the possibility of protease inhibitors penetrating dentin; thus, the application sequence of MMP and catK inhibitors may play an important role in their potential synergistic effect on resisting dentin erosion. However, the information available in the literature is scarce.

Therefore, this study aimed to evaluate the effects of MMP and catK inhibitors on resisting dentin erosion. The following null hypotheses were tested: 1) there was no difference in EDL and the thickness of residual DOM between the protease inhibitors; 2) there was no difference in EDL and the thickness of residual DOM between different application sequences of the protease inhibitors.

## Methodology

The research protocol was approved by the Research Ethics Committee at the School and Hospital of Stomatology, Fujian Medical University (No. 2019Y9030).

### Experimental Design

This *in vitro* study aimed to study the effects of protease inhibitors and their application sequences on EDL and DOM. Specifically, the effect of protease inhibitors was studied at four levels (no inhibition; catK inhibitor; MMP inhibitor; combination of catK and MMP inhibitors). The effect of the application sequence was studied at two levels (inhibition before erosion; inhibition after erosion). Two response variables, EDL and DOM, were studied.

### Sample size calculation

G*Power version 3.1.9.2. for Windows (G*Power, Dusseldorf, Germany) was used to estimate the sample size. The effect size f, α err prob., power, numerator df, and the number of groups were set to 0.4, 0.05, 0.8, 3, and 8, respectively. The results indicated that a minimal specimen number of 10 per group was necessary.

### Preparation of dentin specimens

A total of 56 healthy human third molars that were freshly extracted from 18 to 30 years subjects, male and female, were collected and stored in 0.05% thymol solution at 4°C.^20^ The molars were divided longitudinally into equal halves. Subsequently, separation was performed at the cementum-enamel junction (CEJ) using a low-speed diamond saw (Isomet, Buehler, Lake Bluff, USA) under water irrigation. The coronal enamel was then removed from each half of the tooth at the dentinal-enamel junction (DEJ) to expose the dentin. In total, 96 dentin blocks (3×3×2 mm) were extracted from the central region, located between the pulp horns of the coronal dentin (prepared from 50 molars). A microhardness tester with a Vickers diamond indenter was used to measure the initial microhardness of the dentin surface, ensuring that the dentin blocks were prepared from similar regions. A total of three indentations at 0.3 intervals on the dentin surface were created with a 100 gf load for 10 s.^21^ The average of the three microhardness values within 63.67 to 67.16 kg/mm^2^ was included.^21^ The dentin blocks were further embedded in acrylic resin (Paladur, Heraeus Kulzer, Germany) with a 6 mm internal diameter and a 5 mm height. A baseline surface was finished by polishing the dentin surface using 220-, 600- and 1200-grit silicon carbide abrasive papers and cleaned in an ultrasound bath for two sessions of 1 min. Both sides of the baseline surface were covered with nail varnish (Revlon Corp., NY, USA) to generate a reference surface, and the middle area of the surface (3×1 mm) remained unaltered to receive erosive attacks.^22^ All the specimens were stored in deionized water to maintain humidity.

In addition, six dentin slices (200 μm), parallel to the long axis of the teeth, were cut from six teeth (1 slice/tooth) and used to confirm the presence of MMPs and catK in dentin, as well as to show the enzyme activity before and after treatment with MMP and catK inhibitors.

### Erosive challenges

All 96 specimens were randomly divided into four groups (n=24) according to the following treatment solutions: deionized water (DW, control group, pH=7.3); 1 µM odanacatib (ODN, catK inhibitor, pH=7.5) (Abmole Bioscience, Houston, USA); 1 mM 1,10-phenanthroline (PHEN, MMP inhibitor, pH=7.6) (Abmole Bioscience, Houston, USA); and 1 µM odanacatib + 1 mM 1,10-phenanthroline (COM, combination of catK and MMP inhibitors, pH=7.5/7.6). The specimens in each group were further assigned into two subgroups based on the sequence in which the catK and MMP inhibitors were applied (n=12): before (PRE) and after the erosive challenge (POST).^16^ Both the catK and MMP inhibitors were dissolved in dimethyl sulfoxide (DMSO).

**Figure 1:**
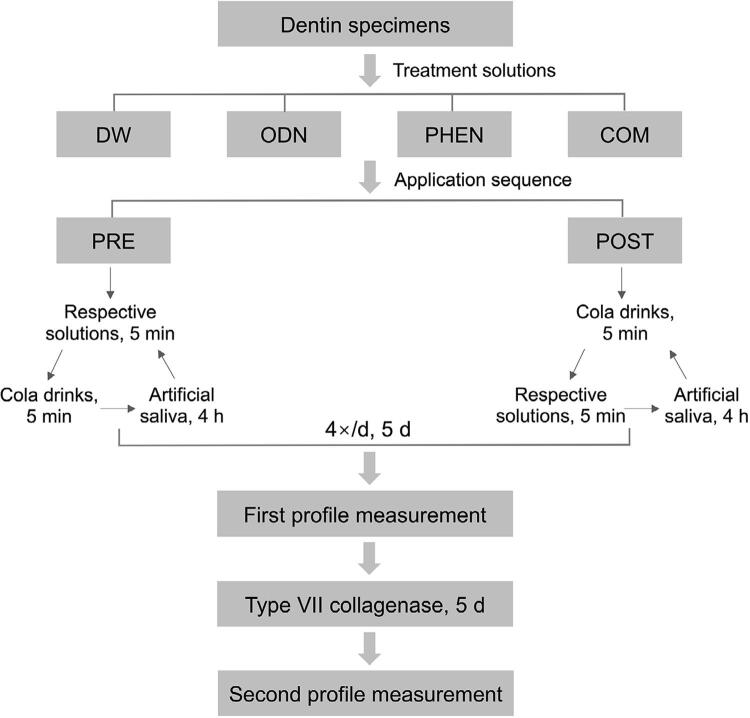
Experimental flowchart

DW: deionized water; ODN: 1 µM odanacatib; PHEN: 1 mM 1,10-phenanthroline; COM: 1 µM odanacatib + 1 mM 1,10-phenanthroline; PRE: before the erosive challenges; POST: after the erosive challenges.

Before the erosive challenges, specimens were first soaked in artificial saliva (containing 0.4 g/l NaCl; 0.795 g/l CaCl_2_·H_2_O; 0.4 g/l KCl; 0.005 g/l Na_2_S·9H_2_O; 0.69 g/l NaH_2_PO_4_·H_2_O; 0.3 g/l KSCN; and 1 g/l urea, pH=6.8) at 37°C for 1 h.^16^ In the subgroup PRE, the specimens were immersed in 50 ml of the respective solutions for 5 min, followed by a 10 s rinse with deionized water.^23^ Subsequently, the specimens were immersed in 150 ml of a cola drink (pH=2.3, Coca Cola, Fuzhou, China) for 5 min and rinsed with deionized water for 10 s;^23^ the cycle was conducted four times (predetermined times at 8, 12, 16, 20 h) per day^24^ for 5 d. Conversely, the specimens in the subgroup POST were first placed in 150 ml of a cola drink for 5 min and then immersed in 50 ml of the different solutions for 5 min. Similarly, a 10 s deionized water rinse was conducted when the acid challenges and solution treatments were finished. Specifically, in the combined treatment with both catK and MMP inhibitors, 1 µM odanacatib was first applied for 5 min, followed immediately by the application of 1 mM 1,10-phenanthroline for 5 min. During the cycling interval and after the last cycle of the day, all specimens were stored in artificial saliva at 37°C (Figure 1). During the experiment, glass containers were used as experimental devices for specimen treatment and storage. Graduated containers were used to control the amount of the treatment solutions, cola drinks, and artificial saliva.

After the 5 d of erosive challenges, two specimens from each subgroup were randomly selected for scanning electron microscopic (SEM) observation. The remaining specimens were used for profile measurement.

### Profile measurement

Nail varnish was carefully removed by a scalpel blade to expose the intact reference surface.^9^ The first profile measurement was performed using a contact profilometer (SEF 680, Kosaka Laboratory, Japan) after the 5 d cycles. The surface of the dentin specimens was scanned using a stylus, the differences in five heights between the reference surface of the specimens and the eroded surface were measured, and the mean value for each specimen was estimated as the EDL (in μm).^5^ During the measurement, the dentin samples were kept moist in deionized water to prevent DOM from shrinking.^4,22^ After that, specimens were incubated in artificial saliva containing 100 U/ml type VII collagenase from Clostridium histolyticum (No. C0773, Sigma-Aldrich, St. Louis, USA) at 37°C for 5 d.^25^ Subsequently, the second profile measurement was conducted. The thickness of residual DOM was quantitatively determined based on the differences in the second and first measurements.^11^

### SEM observation

Of the two specimens selected from each subgroup, half were used to observe the sample surface of the demineralized dentin. The remaining half was fractured into two halves to observe a transverse section of the DOM. The specimens were fixed with 2.5% glutaraldehyde for 4 h and then washed with PBS buffer for 30 min at room temperature. The specimens were dehydrated in an ascending series of ethanol concentrations (30%, 50%, 70%, 80%, 90%, and 100%, for 10 min/concentration) at room temperature.^5,22^ Subsequently, the specimens were dehydrated in a desiccator for 24 h and subjected to gold sputtering. The surface topography and transverse sections of the specimens were observed using a scanning electron microscope (Quanta 450, FEI, USA) with an acceleration voltage of 5 kV. The SEM images were captured at magnifications of 5000× and 2000×.

### Immunolabeled MMP and catK in eroded dentin

A total of two dentin slices that received erosive challenges were used to confirm the presence of MMP-8 and catK in eroded dentin. After incubation with goat serum at 37°C for 30 min, the specimens were incubated at 4°C for 20 h with one of the respective antibodies at 1:100 dilutions: MMP-8 (rabbit anti-human, Abcam, USA) and catK (mouse anti-human, Abcam, USA). The specimens incubated with MMP-8 and catK primary antibodies were then incubated in the dark at 37°C for 1 h with the respective fluorescent secondary antibodies at 1:200 dilutions, respectively (goat anti-rabbit conjugated with Alexa Fluor^®^ 488, Abcam, USA and goat anti-mouse conjugated with Alexa Fluor^®^ 647, Abcam, USA). Phosphate-buffered saline (PBS) was used to wash the excess antibodies during the procedure.

Inverted confocal laser scanning microscopy (CLSM) (TCS SP8, Leica, Germany) was used to observe immunolabeled MMP-8 (excitation/emission: 495/519 nm) and catK (excitation/emission: 655/700 nm) in eroded dentin. Dentin slices were also imaged in the bright field (BF).

### In situ zymography

A total of four dentin slices were used to evaluate the enzyme activity in dentin treated with catK and MMP inhibitors. The dentin slices were etched with 10% phosphoric acid (Scotchbond Etchant, 3M ESPE, Germany) for 10 s.^26^ Then, the dentin slices were rinsed continuously with water for 30 s. Subsequently, four etched dentin slices were treated with 50 μL of the respective treatment solutions for 10 min: DW, ODN, PHEN, and COM.^26^

Fluorogenic dye-quenched (DQ)-gelatin was used *in situ* to evaluate if catK and MMP gelatinolytic activity were inhibited by the catK and MMP inhibitors. Briefly, quenched fluorescein-conjugated gelatin at 1:8 dilutions (E-12055, Molecular Probes, USA) was added to the dentin slices and incubated at 37°C for 24 h, protected from light exposure. CLSM (excitation/emission: 488/530 nm) was used to observe the green fluorescence produced by the dissolved quenched fluorescein-conjugated gelatin mixture produced by MMPs and catK.

### Statistical analysis

The statistical analysis was performed using SPSS software (version 20 for Windows, SPSS, Chicago, IL, USA). The assumptions of normality and equality of variances were confirmed using Kolmogorov-Smirnov and Levene’s tests. Two-way analysis of variance (ANOVA) and Bonferroni’s test were used to assess the effects of MMP and catK inhibitors and their application sequence on EDL and the thickness of residual DOM. Simple effect analysis was adopted when a significant interaction was found.^16^ All statistical analyses were performed at a 0.05 significance level.

## Results

Table 1 shows the EDL and residual DOM of different groups. The different protease inhibitors significantly affected the EDL and thickness of the residual DOM (both p<0.05). The application sequence did not exert a significant effect on the EDL of the tested groups (p>0.05), while the application sequence significantly affected the thickness of residual DOM (p<0.05). Regarding EDL, no significant interaction was found between the different protease inhibitors and application sequences (p=0.613). Regarding DOM, a significant interaction was found between the different protease inhibitors and application sequences (p=0.013).

**Table 1:** Means and standard deviations of the EDL and DOM for different groups (μm)

Group	Description	Subgroup	EDL	DOM
DW	Deionized water	PRE	6.64 (0.87)^a^	1.09 (0.87)^a,b^
		POST	7.36 (1.27)^a^	1.28 (0.74)^a,d^
ODN	CatK inhibitor	PRE	4.20 (1.25)^b^	3.51 (1.61)^c,e^
		POST	4.15 (0.79)^b^	3.19 (1.65)^c,e^
PHEN	MMP inhibitor	PRE	4.41 (1.38)^b^	2.62 (1.04)^a,c^
		POST	5.13 (1.80)^b^	1.12 (0.73)^b,d^
COM	Combination of catK and MMP inhibitors	PRE	4.31 (1.04)^b^	2.61 (1.02)^a,c^
		POST	4.16 (1.95)^b^	3.88 (2.14)^e^

Different lowercase letters in a column indicate significant differences in different groups and subgroups (P<0.05).

DW: deionized water; ODN: 1 µM odanacatib; PHEN: 1 mM 1,10-phenanthroline; COM: 1 µM odanacatib + 1 mM 1,10-phenanthroline; PRE, before the erosive challenges; POST, after the erosive challenges.

Significantly less EDL was observed in the groups ODN, PHEN, and COM than in the group DW (all p<0.05). No significant differences in EDL were detected among the groups ODN, PHEN, and COM (all p>0.05). A significantly thicker DOM was observed in the groups ODN and COM than in the group DW (both p<0.05). No significant differences were found in the DOM between the groups PHEN and DW (p=0.615).

A simple effect analysis was adopted since a significant interaction was found between the different protease inhibitors and application sequences (p=0.013) regarding DOM. In the subgroup PRE, ODN showed a significantly thicker DOM than that of the control group (p=0.001). In the subgroup POST, both ODN and COM preserved a significantly thicker DOM than that of the control group (both p<0.05). No significant differences in DOM were observed between the subgroups PRE and POST for ODN (p=0.588). Regarding COM, the subgroup POST preserved a significantly thicker DOM than that of the subgroup PRE (p=0.034).

**Figure 2:**
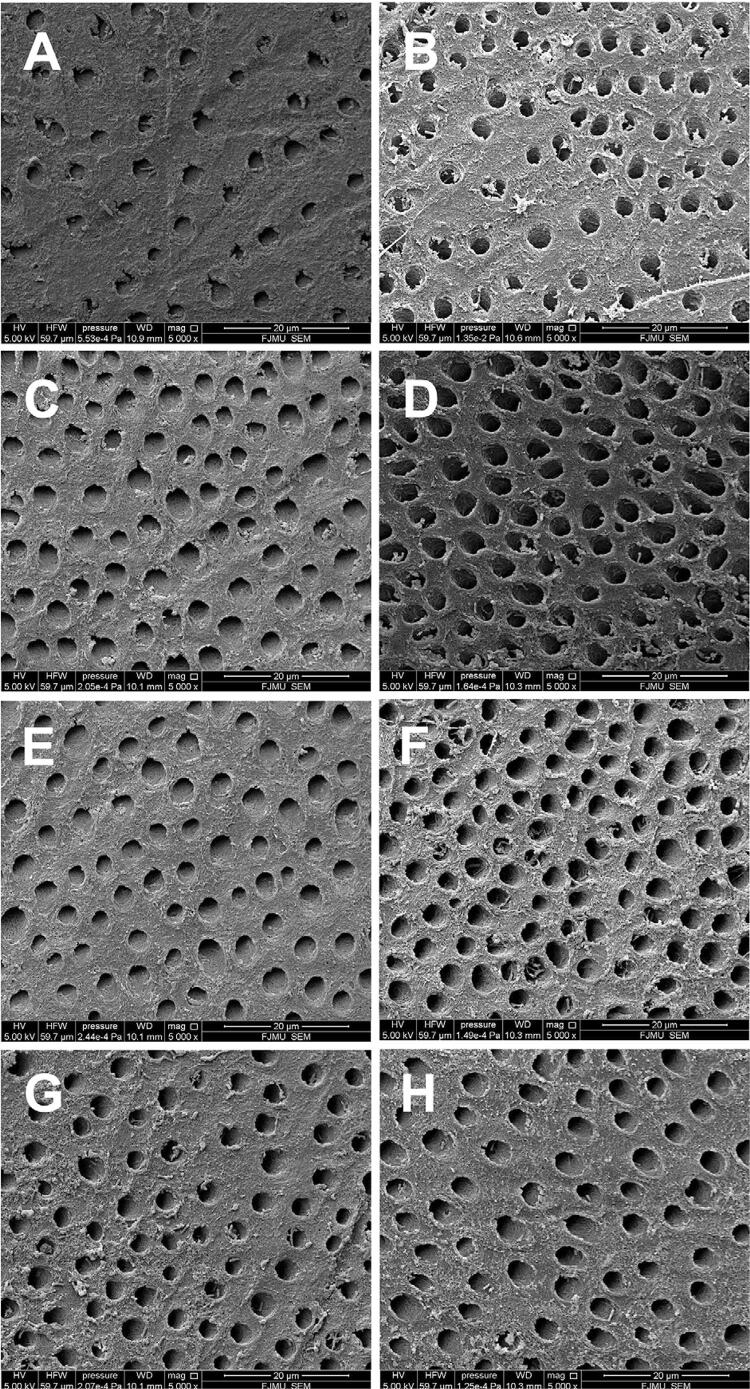
Representative SEM images (×5000) of the specimens (dentin surface) treated with the respective solutions in subgroups PRE (A, C, E, G) and POST (B, D, F, H)

**Figure 3:**
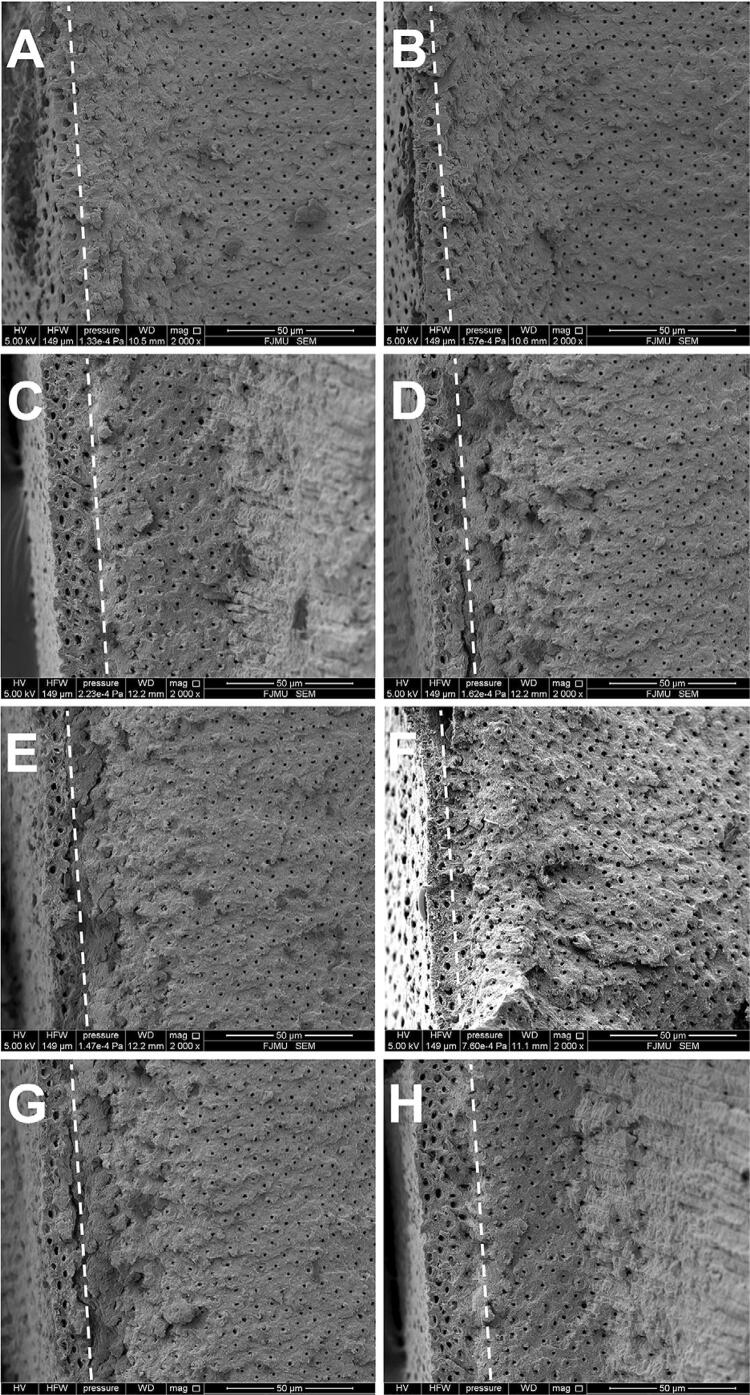
Representative SEM images (×2000) of the specimens (transverse sections) treated with the respective solutions in subgroups PRE (A, C, E, G) and POST (B, D, F, H).

SEM images showed that the dentinal tubules were wider in the groups ODN, PHEN, and COM than in the control group. Moreover, the subgroups POST showed more numerous and wider dentinal tubules than the subgroups PRE for all groups (Figure 2). On the transverse section, thicker DOM was observed in the groups ODN and COM (Figure 3).

A, B: specimen treated with deionized water; C, D: specimen treated with 1 µM odanacatib; E, F: specimen treated with 1 mM 1,10-phenanthroline; G, H: specimen treated with 1 µM odanacatib + 1 mM 1,10-phenanthroline.

A, B: specimen treated with deionized water; C, D: specimen treated with 1 µM odanacatib; E, F: specimen treated with 1 mM 1,10-phenanthroline; G, H: specimen treated with 1 µM odanacatib + 1 mM 1,10-phenanthroline. The demineralized organic matrix (DOM) is indicated by the dashed line.

**Figure 4:**
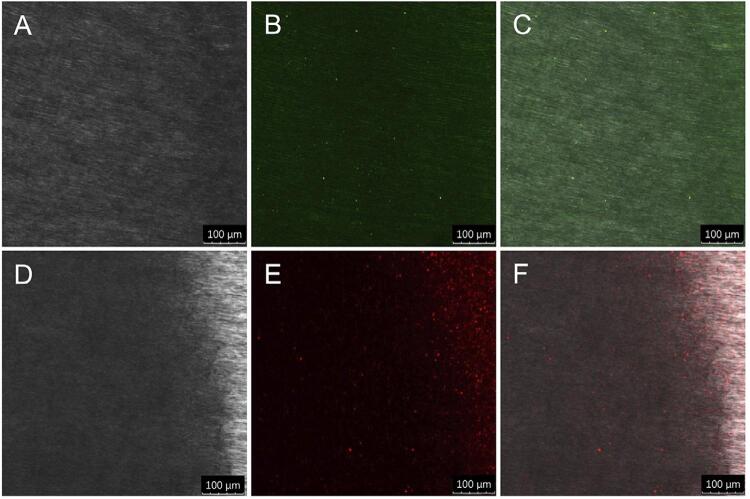
CLSM images of immunolabeled MMP-8 (A, B, C) and catK (D, E, F) in eroded dentin

**Figure 5:**
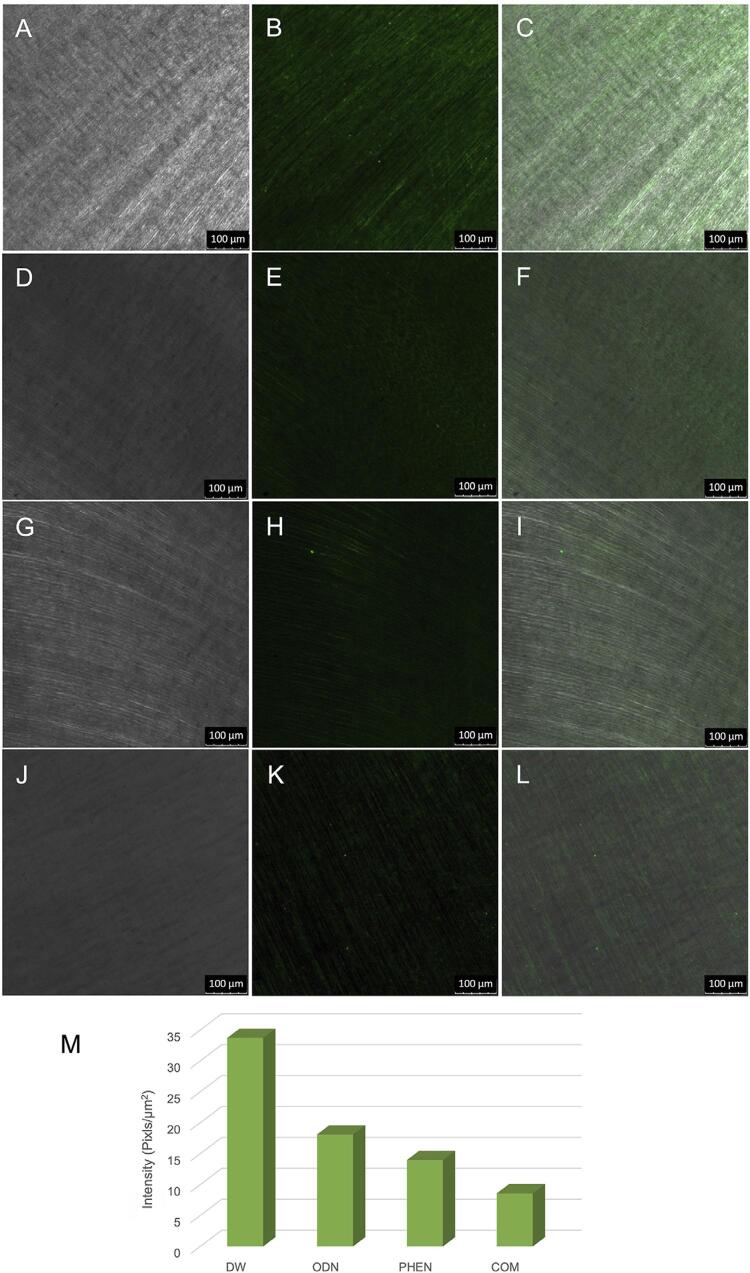
CLSM images of the in situ zymography of the dentin slices without (A, B, C) or with (D, E, F, G, H, I, J, K, L) inhibitory treatment after incubation for 24 h

CLSM images indicated the presence of MMP-8 (Figure 4 – B and C) and catK (Figure 4 – E and F) in eroded dentin. *In situ* zymography showed that the gelatinolytic activities of catK and MMP were inhibited by ODN and PHEN. For the treatment with DW, an intense green fluorescence indicated gelatinolytic activity in dentin without inhibitory treatment. Treatment with ODN, PHEN, and the combination of ODN and PHEN inhibited the gelatinolytic activity by approximately 46.32%, 58.6%, and 74.56%, respectively, as shown by the decrease in green fluorescence (Figure 5).

A, D: bright field, showing the optical density of eroded dentin surface; B: acquired in green channel, indicating immunolabeled MMP-8 in eroded dentin; E: acquired in red channel, indicating immunolabeled catK in eroded dentin; C, F: merged images.

A, D, G, J: bright field, showing the optical density of the dentin surface; B, E, H, K: acquired in green channel, showing fluorescence in dentin; C, F, I, L: merged images. A-C: dentin slices treated with deionized water (DW, without inhibitory treatment); D-F: dentin slices treated with 1 µM odanacatib (ODN); G-I: dentin slices treated with 1 mM 1,10-phenanthroline (PHEN); J-L: dentin slices treated with 1 µM odanacatib + 1 mM 1,10-phenanthroline (COM). M: quantified green fluorescence of the specimens with different treatments. The intense green fluorescence indicated strong gelatinolytic activity in dentin without inhibitory treatment. Treatment with catK and MMP inhibitors led to decreased green fluorescence, indicating reduced gelatinolytic activity after inhibitory treatment.

## Discussion

Compared to enamel erosion, dentin erosion progresses much faster due to differences in mineral content and impurity, as well as the enzymatic degradation of collagen by endogenous MMPs and catK in dentin.^5,19,27^ To the authors’ knowledge, this study was the first to investigate the synergistic effects of MMP and catK inhibitors on resisting dentin erosion by considering the application sequence. Based on our findings, the null hypotheses of no difference in EDL between the protease inhibitors and no difference in EDL between the application sequence of protease inhibitors were accepted. The null hypotheses of no difference in the thickness of residual DOM between the protease inhibitors and no difference in the thickness of residual DOM between application sequences of protease inhibitors were rejected. The research protocol, specifically the regimen of erosive challenges, the application times, and the concentrations of the tested inhibitors were determined according to previous studies.^5,14,16,23^ In addition, among all known CCs, catK is the only one with triple helical collagenase activity^14^ and it has been indicated that pro-MMP-9 can be activated by catK.^13^ Therefore, we used ODN in this study because it is a specific inhibitor of catK.^25^

The application of MMP and catK inhibitors alone significantly reduced EDL, agreeing with previous studies.^5,11^ Moreover, the application sequence did not compromise the effectiveness of MMP and catK inhibitors in reducing EDL. MMPs are a family of Zn^2^- and Ca^2^-dependent enzymes, and PHEN may inactivate MMPs by chelating Zn^2^ with two N atoms.^12,28^ Furthermore, PHEN is a small-molecule^29^ (molecular weight: 180.205) metal chelator;^30,31^ thus, PHEN may remain in dentin and function regardless of the condition in which it was applied. Pertinently, catK is a cysteine proteinase, containing a thiol group (-SH).^32^ ODN may combine with the -SH of catK and thus prevent catK from binding to its substrates.^32^ The optimal pH of catK has a short duration, which may explain why catK inhibitors exhibited a similar effect when applied before and after erosion.^11^ Interestingly, no synergistic effect of MMP and catK inhibitors on dentin erosion was detected in this study. Activated MMPs that are induced by catK may further activate other proMMPs; thus, catK may play an important role in the cascade reaction of MMPs.^12,13,33,34^ As a result, inhibiting catK might greatly influence the cascade reaction of MMPs, resulting in a similar EDL to that obtained when inhibiting MMPs individually. However, this hypothesis should be clarified in future studies. Despite the similar EDL in the subgroups PRE and POST, treatment with MMP and catK inhibitors after erosive challenges showed more numerous and wider dentinal tubules than that before erosive challenges, which might indicate more aggressive acid etching.

Interestingly, a similar DOM thickness was observed in the group treated with the MMP inhibitor and in the control group, which is in line with the findings from Zarella, et al.^11^ (2015). However, contradictory results were reported by Yang, et al.^5^ (2022). The contrasting results may be related to the different study protocols (e.g., erosion duration, application time, and types of inhibitors). MMPs lead to excessive DOM degradation after exposure to an acidic pH for a sufficient duration and neutralization by saliva buffer.^11^ With our protocol, MMPs may be insufficiently inhibited since the concentration of inhibitor necessary to effectively inhibit enzymatic activity is related to the application method (e.g., stronger inhibition occurred when MMP inhibitor was mixed with MMPs than when added to zymography buffer).^8^ Applying the catK inhibitor and the combination of the two inhibitors significantly preserved a thicker DOM than that of the control group. In addition, a significantly thicker DOM was observed when the combined application was performed after erosion rather than before erosion. Compared to treatment with an MMP inhibitor before erosion, higher MMP activity (11-17% higher) was detected when dentin specimens were treated with an MMP inhibitor following acid erosion.^8^ As mentioned earlier, it is possible that catK plays an essential role in the cascade reaction of MMP activation.^12,13,34^ Hence, although the collagen degradation by catK itself was seemingly weak, inhibiting catK achieved an EDL similar to that obtained by MMP inhibition and even preserved a thicker DOM. Pertinently, the role of catK in DOM degradation deserves further research. It is noteworthy that the solvent of the catK and MMP inhibitors used in this study, namely, DMSO, is not inert. In fact, DMSO is an effective surfactant and enhances penetration into dentin.^35^ Moreover, DMSO may have an impact on dentin enzyme activity, thus decreasing collagen degradation.^36,37^ Accordingly, these effects might contribute to our results, and further research on the effects of DMSO on enzyme activity and DOM degradation in dentin erosion would be interesting.

However, no obvious relationship between DOM retention and EDL was found either in this or in previous studies.^11^ DOM is regarded as resistant to abrasive forces and beneficial to dentin remineralization.^6,8,38^ In an erosion/abrasion study, Ganss, et al.^6^ (2009) reported that DOM was present on all samples loaded with the brushing force (2-4 N), although it was compressed. The striking resistance of DOM to abrasive forces may be related to its remarkable tensile properties.^6^ Furthermore, the presence of DOM promoted the fluoride effect, promoting the remineralization of erosive dentin, reducing the cumulative mineral loss by approximately 39-73%; however, in the absence of DOM, there was a significant increase in mineral loss (compared to that of the control group), even though fluoride was applied in association with the remineralization solution.^38^ Given the differences in the experiment conditions mentioned above, it is credible that acquiring thicker DOM is of significant importance under certain conditions. Remineralization can take place based on DOM, especially in the presence of calcium, phosphate, and fluoride.^8^ Keeping DOM intact and reproducing the dimensions and structural hierarchy of apatite deposits within the DOM is vital for making it remineralizable.^39^

Based on our findings, applying MMP and catK inhibitors is an effective method for reducing dentin erosion. An MMP or catK inhibitor can be applied before or after erosion, but the combined application is not strictly necessary. CatK inhibitors are considered a potentially promising solution for managing dentin erosion. The products tested in this study were all analytical-grade reagents (high purity) with acceptable costs. For clinical usage, it will be necessary to further identify a reasonable carrier for MMP and catK inhibitors, such as a gel or paste.

A limitation of this *in vitro* study is that the real state of the oral environment cannot be represented. Moreover, only one concentration and duration of protease inhibitors were tested. No positive control was adopted since the study was performed to provide a proof of concept for the synergistic effects of MMP and catK inhibitors on dentin erosion. Commonly used anti-erosive agents, such as stannous fluoride and sodium fluoride, should be included as positive controls in further studies. In addition, further clinical studies are needed to confirm our findings.

## Conclusions

Within the limitations of this study, the following conclusions can be prudently drawn:

1. The inhibition of endogenous dentinal MMPs and catK increases the acid resistance of human dentin but shows no apparent synergistic effect.
2. The inhibition of MMPs and catK either before or after the acid challenge is equally effective.
3. The inhibition of catK seems to be the simplest approach and least dependent on the application mode when the inhibition of endogenous dentinal collagenolytic enzymes is considered in the prevention of dentin erosion progression.
